# Current Challenges in Elucidating ALS Disease Mechanisms and Therapeutic Advances

**DOI:** 10.3390/cells14100715

**Published:** 2025-05-14

**Authors:** Smita Saxena, W. David Arnold

**Affiliations:** 1 Department of Physical Medicine and Rehabilitation, School of Medicine, University of Missouri, Columbia, MO 65211, USA; wdavidarnold@health.missouri.edu; 2 NextGen Precision Health, University of Missouri, Columbia, MO 65211, USA

As a researcher and a physician working together to combat amyotrophic lateral sclerosis (ALS), we are acutely aware of both the urgent need for innovation and the persistent divide between laboratory discoveries and clinical care. Too often, promising scientific advances fail to reach patients in time. In a disease like ALS, where progression is rapid and the therapeutic window is narrow, this disconnect carries real consequences [1]. Our work at NexGen Precision Health is driven by the belief that integrating research and medicine is not only possible, but essential. A central tenet of precision medicine is delivering the right treatment to the right patient at the right time, which can only be achieved by understanding the underlying biology of each ALS subtype. Genomics, proteomics, and biofluid biomarkers are now making it possible to classify patients more accurately, detect targetable changes earlier, and tailor therapies accordingly. This is especially critical in ALS, where time is the most valuable resource we have. This Special Issue of *Cells* highlights both the challenges and opportunities in ALS research, from the role of microglia and Schwann cells to the impact of aging, metabolism, and circuit dysfunction. It also underscores the need for better biomarkers, novel therapeutic targets, and a collaborative infrastructure to turn discoveries into durable clinical gains.

Both ALS and FTD are now recognized as a disease spectrum, sharing an overlapping pathology, including protein aggregation and selective neuronal degeneration. While familial ALS (fALS) is linked to over 50 genes, including *SOD1*, *C9ORF72*, *FUS*, and *TARDBP*, the cause of sporadic ALS remains largely unknown, though genetic and environmental factors contribute [2]. Traditionally considered a neuron-centered disease, growing evidence highlights the role of non-neuronal cells in both neurotoxicity and neuroprotection. **Bond et al**. (contribution 1) explore the role of microglia in ALS, emphasizing their contributions to motor neuron degeneration and discussing emerging therapies that target microglial function. **Moss et al**. (contribution 2) highlights the underexplored role of Schwann cells in ALS, drawing parallels to Charcot–Marie–Tooth disease and suggesting their potential as therapeutic targets for nerve repair. **Salzinger et al.** (contribution 3) examine how neuronal circuit dysfunction contributes to ALS pathogenesis. By integrating findings from various preclinical models, they provide insights into upper motor neuron degeneration, lower motor neuron hyperexcitability, and the neuromuscular junction’s deterioration [1]. Environmental and aging-related factors in ALS are explored by Hernan-Godoy et al. and Dashtmian et al. **Hernan-Godoy et al.** (contribution 4) investigate the role of one-carbon metabolism alterations and epigenetic dysregulation, debating whether these arise from lifelong environmental exposure, late-stage triggers, or early developmental defects. **Dashtmian et al.** (contribution 5) examine the convergence between age-related muscle decline and ALS pathology, proposing senolytic therapies as a potential avenue to reduce neuroinflammation and slow disease progression. Moving towards mechanistic implications, **Motaln et al.** (contribution 6) review the role of *c-Abl* in neurodegeneration, focusing on its interactions with stress signaling, DNA damage, and metabolism. **Smeele et al.** (contribution 7) discuss dipeptide repeat proteins (DPRs) in *C9ORF72*-linked ALS/FTD, emphasizing their toxicity, aggregation, and therapeutic implications.

Therapeutic advancements are further explored by **Lundt et al.** (contribution 8), who review strategies targeting NAD+ metabolism, including NAD+ precursors and enzyme modulators that show promise in ALS models. **Nguyen L.** (contribution 9) provides an update on ALS therapies, including the 2023 FDA-approved antisense oligonucleotide (ASO) drug for *SOD1* ALS, new insights into *C9ORF72* repeat expansion, and emerging biomarkers for diagnostics and treatment. **Bagyinszky et al.** (contribution 10) focus on proteomic biomarkers, microRNAs, and extracellular vesicles as accessible tools for disease monitoring and early risk assessment, providing new avenues for biomarkers and translational research.

Nevertheless, developing effective ALS therapies remains challenging. The disease’s heterogeneity, involving both genetic and environmental factors, complicates treatment development. Late diagnosis limits intervention effectiveness, and the disease progresses rapidly, narrowing the treatment window, while the blood–brain barrier (BBB) restricts drug delivery to motor neurons. Clinical trials face obstacles due to patient variability and small sample sizes [3,4]. At the University of Missouri, we are working to change this through the NextGen Precision Health initiative, an interdisciplinary effort designed to streamline the path from discovery to clinical application. By co-locating research laboratories, clinical investigators, and computational scientists under one roof, we are building a shared infrastructure that supports rapid iteration and collaborative translational science. This model enables earlier diagnosis, improves clinical trial design, and supports real-time monitoring of therapeutic response, all anchored around patient-centered goals. Recognizing the complexity of ALS, we have built a team that reflects the multifaceted nature of the disease. ALS is increasingly understood not as a single disorder, but as a spectrum of overlapping syndromes unified by motor neuron degeneration. This heterogeneity, genetic, biological, and clinical, demands precision approaches [5,6].

Thus, our goal is that personalized medicine approaches will ultimately enhance therapeutic efficacy, reduce adverse effects, and enable earlier interventions. In complex and heterogeneous diseases like ALS, precision medicine would help in identifying disease subtypes, uncover novel drug targets, and accelerate the development of targeted therapies—ultimately improving outcomes and paving the way for more effective and patient-specific care.
